# Food Proteins: Processing, Interactions, Functionality and Bioavailability

**DOI:** 10.3390/foods14050857

**Published:** 2025-03-03

**Authors:** Mike Boland, Lovedeep Kaur, Alejandra Acevedo-Fani

**Affiliations:** 1 Riddet Institute, Massey University, Private Bag 11222, Palmerston North 4442, New Zealand; l.kaur@massey.ac.nz (L.K.); a.acevedo-fani@massey.ac.nz (A.A.-F.); 2 School of Food and Advanced Technology, Massey University, Private Bag 11222, Palmerston North 4442, New Zealand

Protein is an essential part of our diet. As the world population increases, and pressure on the environment from farming grows, there is a need for more efficient and greener methods of production and processing; there is also a need to grow the production of alternative proteins that can supplement or partially replace the existing protein supply, particularly with respect to animal-derived proteins [1]. The landscape of food protein is changing due to the emergence of novel proteins through genetic technologies and from new methods of processing that can change their nature.

Proteins in foods perform two main functions: nutrition and modification of physical functionality. Protein nutrition is a function of the amino acid composition of the protein and the bioavailability of the dietary essential amino acids. In addition to this, many proteins, when digested, produce peptides that are bioactive. This bioactivity can be positive, such as peptides from milk proteins that enhance calcium uptake or lower blood pressure, or it can be negative, such as the peptides that cause gluten intolerance.

Physical functionality includes such factors as water binding, fiber formation, gel formation, and emulsification. These functionalities can affect the sensorial properties of food and can be modified by changing the structure of the proteins, for example by denaturation. These changes can, in turn, change the way a protein is digested (or not digested), changing its nutritional and bioactive properties.

In this Special Issue, we discuss progress in these areas and the potential impact of novel processes and proteins on the nutritional and health properties of these proteins.

The paper from Zhu et al. (Contribution 1) explores the properties of proteins and peptides extracted from bovine bone as emulsifiers, and a function of molecular weight, giving scope for the development of novel ingredients from this source, which is often wasted. The peptides (and small proteins) were separated by ultrafiltration, and a fraction with MW of 10–30 kDa shows particular promise.

Goat milk whey is a by-product of the goat cheese industry, and, unlike cows’ milk, whey, is relatively undeveloped as a source of protein ingredients. The paper by Tian et al. (Contribution 2) explores the effect of thermally-induced polymerization on a range of functional properties of goat milk whey protein.

Novel processes have opened the way to developing new and different functional properties of many proteins.

An important emerging process is high-moisture extrusion of plant proteins to develop fibrous structures that mimic meat products. This is reviewed in depth by Sengar et al. (Contribution 3). Importantly, the different effects of heat and shear during extrusion are described, and their potential for making different kinds of protein products is explored.

The paper by Periera et al. (Contribution 4) reviews the use of direct electric field technologies on the processing food proteins. The authors discuss Pulsed Electric Fields (PEF), Moderate Electric Fields, Ohmic heating (OH), Pulsed Ohmic Heating (POH), and High-Voltage Electric Field Discharge (HVED), focusing on their potential to modify conventional and alternative proteins at a macrostructural and nanostructural level. These modifications have important implications for the functional properties of proteins and in the development of novel protein structures with potential benefits extending to the biomedical and pharmaceutical industries.

Finally, the review by Ajomiwe et al. (Contribution 5) looks at various sources of protein and their importance for human nutrition, specifically examining their structure, digestibility, and bioavailability. It compares the nutritional value of animal proteins with those of plant-based sources like legumes, nuts, and seeds and also discusses novel proteins such as cultured meat, insect proteins, and single-cell proteins, considering their nutritional value and potential allergenicity.
